# Impact of medicaid expansion on disparities in revascularization in patients hospitalized with acute myocardial infarction

**DOI:** 10.1371/journal.pone.0243385

**Published:** 2020-12-23

**Authors:** Laurent G. Glance, Caroline P. Thirukumaran, Ernie Shippey, Stewart J. Lustik, Andrew W. Dick

**Affiliations:** 1 Department of Anesthesiology and Perioperative Medicine, University of Rochester School of Medicine, Rochester, NY, United States of America; 2 Department of Public Health Sciences, University of Rochester School of Medicine, Rochester, NY, United States of America; 3 RAND Health, RAND, Boston, MA, United States of America; 4 Department of Orthopedics, University of Rochester School of Medicine, Rochester, NY, United States of America; 5 Research Analyst, Vizient, Irving, TX, United States of America; Case Western Reserve University School of Medicine, UNITED STATES

## Abstract

**Introduction:**

Blacks are more likely to live in poverty and be uninsured, and are less likely to undergo revascularization after am acute myocardial infarction compared to whites. The objective of this study was to determine whether Medicaid expansion was associated with a reduction in revascularization disparities in patients admitted with an acute myocardial infarction.

**Methods:**

Retrospective analysis study using data (2010–2018) from hospitals participating in the University Health Systems Consortium, now renamed the Vizient Clinical Database. Comparative interrupted time series analysis was used to compare changes in the use of revascularization therapies (PCI and CABG) in white versus non-Hispanic black patients hospitalized with either ST-segment elevation (STEMI) or non-ST-segment elevation acute myocardial infarctions (NSTEMI) after Medicaid expansion.

**Results:**

The analytic cohort included 68,610 STEMI and 127,378 NSTEMI patients. The percentage point decrease in the uninsured rate for STEMIs and NSTEMIs was greater for blacks in expansion states compared to whites in expansion states. For patients with STEMIs, differences in black versus white revascularization rates decreased by 2.09 percentage points per year (95% CI, 0.29–3.88, P = 0.023) in expansion versus non-expansion states after adjusting for patient and hospital characteristics. Black patients hospitalized with STEMI in non-expansion states experienced a 7.24 percentage point increase in revascularization rate in 2014 (95% CI, 2.83–11.7, P < 0.001) but did not experience significant annual percentage point increases in the rate of revascularization in subsequent years (1.52; 95% CI, -0.51–3.55, P = 0.14) compared to whites in non-expansion states. Medicaid expansion was not associated with changes in the revascularization rate for either blacks or whites hospitalized with NSTEMIs.

**Conclusion:**

Medicaid expansion was associated with greater reductions in the number of uninsured blacks compared to uninsured whites. Medicaid expansion was not associated, however, with a reduction in revascularization disparities between black and white patients admitted with acute myocardial infarctions.

## Introduction

Cardiovascular disease remains the number one cause of death in the U.S. [1] and disproportionately affects racial minority and low-income populations [2]. Black males have the shortest life expectancy compared to all other racial and ethnic groups [3,4], and cardiovascular disease is responsible for one-third of the mortality disparity between blacks and whites [5,6]. Despite the proven effectiveness of cardiovascular procedures to improve survival, black patients with coronary heart disease are less likely to undergo cardiac catheterization, percutaneous coronary intervention, or surgical revascularization [7–11].

Lack of health insurance may be an important cause of racial disparities in cardiovascular health. Uninsured patients with cardiovascular disease receive less aggressive care and have worse outcomes [12]. The Patient Protection and Affordable Care Act (ACA) was intended to improve access to health care and outcomes in previously uninsured patients by expanding Medicaid, creating health insurance marketplaces, eliminating exclusions for pre-existing conditions, and providing income-based subsidies [13]. Medicaid expansion was associated with reductions uninsured hospitalizations for cardiovascular events [14], a lower rate of cardiovascular death [15], higher rates of cancer surgery [16], and more optimal management of serious surgical problems [17]. Overall, Medicaid expansion is associated with a 6% reduction in mortality rate in the non-elderly population, or 20 fewer deaths per 100,000 at a cost between $327,000 to $867,000 per life saved [18].

Prior to the implementation of the ACA, 25.8% of non-elderly blacks lacked health insurance compared to 14.8% of whites [19]. Because uninsured rates dropped more for blacks in states that expanded Medicaid access compared to states that did not expand Medicaid access [20], we hypothesized that Medicaid expansion may have led to reductions in disparities between blacks and non-Hispanic whites in cardiovascular care. Since guideline-based therapies in AMI patients are both effective and resource-intensive, and racial disparities in the use of these therapies are pervasive and persistent, we performed a retrospective cohort quasi-experimental analysis to examine whether Medicaid expansion was associated with reductions in racial disparities in the use of invasive therapies (percutaneous coronary intervention and coronary artery bypass grafting) in patients hospitalized with AMI in major academic medical centers and their affiliates. Because insurance coverage increased in all states after the ACA was adopted, we also examined the association between the ACA and disparities in the use of invasive therapies in all states, regardless of whether they expanded Medicaid access. Since the American Heart Association/American College of Cardiology guidelines in 2007 and 2014 recommend early invasive therapy for ST-segment elevation myocardial infarctions [21,22], while allowing both early invasive therapy or ischemia-guided therapy for non-ST segment elevation myocardial infarctions [23,24], we conducted separate analyses for ST and non-ST segment elevation myocardial infarctions. Understanding the impact of the ACA on disparities in cardiovascular care is important given current efforts to repeal the ACA, and the potential to increase the number of the uninsured by 18 million in the first year of repeal [13].

## Methods

### Data source

This retrospective cohort study of adult non-elderly patients hospitalized with either non-ST-segment elevation acute myocardial infarctions (NSTEMI) or ST-segment elevation acute myocardial infarctions (STEMI) was based on data (2010–2018) from the Vizient Clinical Database/Resource (CDB/RM), formerly known as the University HealthSystem Consortium (UHC). The University HealthSystem Consortium is an alliance of 117 academic medical centers and their affiliated hospitals [25], and includes over 90% of academic medical centers in the U.S. [17]. These data have been used in multiple prior studies [17,26–28]. The database includes information on patient demographic characteristics, admission source, International Classification of Diseases (ICD), Ninth and Tenth Revision, diagnostic and procedure codes, All Patients Refined Diagnosis Related Groups Severity of Illness (APR-DRG SOI) and Risk of Mortality (APR-DRG ROM), encrypted hospital identifiers, and state identifiers. The Institutional Review Board of the University of Rochester’s School of Medicine (Rochester, New York) reviewed and approved this study. The requirement for informed consent was waived because of the retrospective nature of this study, this research involved no more than minimal risk to the subjects, and this research could not practicably be carried out without the waiver. The STROBE recommendations [29] were used to guide the reporting of this study.

### Study population

We identified 310,661 admissions for NSTEMI and 153,820 admissions for STEMI (flow diagrams are shown in S1 Appendix Fig 1A and 1B) in patients between the ages of 18 and 64. STEMIs and NSTEMIs were identified using ICD9-CM and ICD10-CM codes (S1 Appendix Table 1). We excluded patients 65 years and older because these patients were not eligible for Medicaid Expansion under the ACA [17]. Because a new ICD-10CM code for type 2 myocardial infarction (demand ischemia) (I21.A1) was coded in 2018, and this code was coincident with a significant drop in NSTEMIs but not STEMIs in our data set, we excluded 2018 from our NSTEMI analysis. Records with missing information on race (STEMI 4,732; NSTEMI 6,567) and sex (STEMI 13, NSTEMI 7) were excluded. The study was limited to whites and non-Hispanic blacks, leading to the exclusion of 23,196 and 44,197 records for STEMIs and NSTEMIs, respectively. Medicare patients were also excluded (STEMI, 12,397; NSTEMI 48,096). The analysis was limited to hospitals that spanned the study period and did not include states that expanded Medicaid access after 2014 (STEMI 10,650; NSTEMI 21,410). The final analytic data set consisted of 68,610 STEMIs and 127,378 NSTEMIs from 175 hospitals.

### Analysis

The main outcome variable was a composite of the use of coronary artery bypass grafting (CABG) or percutaneous coronary intervention (PCI) during the index hospitalization, defined using ICD9-CM and ICD10-PCS codes (S1 Appendix Table 1). Our primary hypothesis is that Medicaid expansion was associated with a reduction in the black-white disparity in the use of revascularization. We compared patients residing in states that expanded Medicaid access in 2014 to patients residing in states that did not expand Medicaid access (S1 Appendix Tables 3 and 4).

To evaluate the association between Medicaid expansion and racial disparities, we estimated a comparative interrupted time series (CITS) model [30] which is similar to, but more flexible than a classic difference-in-difference model [31]. The classic difference-in-difference approach can be used to control for differences between patients hospitalized in non-expansion states and expansion states [31], as long as the trends in revascularization for these 2 groups in the pre-intervention period (before and after 2014) are parallel. The CITS approach accommodates different pre-intervention utilization time-trends across groups, thus controlling for potential differences in revascularization rates between expansion and non-expansion states even when the pre-expansion trends are not parallel.

We specified the CITS model as a linear regression so that the intervention effects could be interpreted as the absolute changes in the rate of revascularization Y_iht_ for patient *i* in year *t* in hospital *h* (model 1) [30]:

$$\begin{array}{l} E\left(Y_{ith}\right)=\beta_{1}+\beta_{2}Black_{ith}+\beta_{3}ME_{}+\beta_{4}Black_{ith}\;x\;ME+\\ \;(\beta_{5}Year_{t}+\beta_{6}Year_{t}\;x\;ME+\beta_{7}Year_{t}\;x\;Black_{ith}+\beta_{8}Year_{t}\;x\;Black_{ith}\;x\;ME)+\\ \;(\beta_{9}Post+\beta_{10}Post\;x\;ME+\beta_{11}Post\;x\;Black_{ith}+\beta_{12}Post\;x\;Black_{ith}\;x\;ME)+\\ \;(\beta_{13}PostYear_{t}+\beta_{14}PostYear_{t}\;x\;ME+\beta_{15}PostYear_{t}\;x\;Black_{ith}+\beta_{16}PostYear_{t}\;x\;Black_{ith}\;x\;ME)+\\ \;\beta_{17q}Season_{q}+\beta_{18p}Covariates_{ith(p)}+\beta_{19h}H_{h} \end{array}$$

We controlled for the common pre-expansion time trend *Year*_*t*_, baseline differences between expansion and non-expansion states (ME), and baseline differences between whites and non-Hispanic blacks. We controlled for hospital characteristics using hospital fixed effects (H_h_) to control for the possibility that hospitals in expansion states differed from hospitals in non-expansion states. We specified interaction terms that allowed the pre-intervention trends to differ between whites and blacks in expansion and non-expansion states (β_6_, β_7_ and β_8_). We specified an intercept shift (*Post*) and a separate linear post-expansion time trend (PostYear_t_) to control for changes in the post-intervention period. We included interaction terms for the intercept shift (β_10_, β_11_, and β_12_) and post-expansion time trend (β_14_, β_15_, and β_16_) between whites and blacks in expansion and non-expansion states. We adjusted for patient-level covariates, including age, sex, transfer from outside institution, urgency of admission, history of prior cardiac surgery or PCI, APR-DRG Severity-of-Illness, APR-DRG Risk-of-Mortality, and comorbidities coded using the Elixhauser comorbidity algorithm [32,33]. The estimated coefficient β_11_ characterizes the intercept change for blacks living in non-expansion states in 2014 relative to whites in non-expansion states. β_16_ represents the difference in black versus white revascularization rate trends in expansion versus non-expansion states.

We used this model to estimate average marginal effects for blacks and whites during the pre and post-intervention period in expansion and non-expansion states. We used cluster robust variance estimators to account for clustering of outcomes within hospitals, autocorrelation of repeated measures, and heteroskedastic error terms. Separate analyses were conducted for STEMIs and NSTEMIs. Because we considered each of these analyses to address separate questions, we did not adjust for multiple comparisons.

### Secondary analyses

We also estimated regression discontinuity models to examine the association between the Affordable Care Act and (1) the overall revascularization rate, and (2) in-hospital mortality. The mortality models were specified as logistic regressions because linear models may lead to mis-specification when binary outcomes occur with low frequency. We used the period before 2014 to identify the pre-ACA period because two of the major components of the ACA, Medicaid expansion and the creation of health insurance exchanges, were implemented in 2014 [34].

We performed several sensitivity analyses. First, we estimated separate interrupted time series model in which year *T*_*t*_ was specified as separate indicator variables (in contrast to the CITS model where year was specified as a continuous variable) over the entire study period, also controlling for season, patient covariates, and hospital fixed effects:

$$\begin{array}{l} E\left(Y_{ith}\right)=\alpha_{0}+\alpha_{1}Black_{ith}+\alpha_{2}ME+\alpha_{3}Black_{ith}\;x\;ME+\\ \;\alpha_{4t}T_{t}+\alpha_{5t}T_{t}\;x\;ME+\alpha_{6t}T_{t}\;x\;ME\;x\;Black_{ith}+\\ \;\alpha_{7q}Season_{q}+\alpha_{8p}Covariates_{ith(p)}+\alpha_{9h}H_{h} \end{array}$$

This model allowed us to estimate average marginal effects, after adjusting for patient-level and hospital effects, without imposing any linear restrictions on rates of revascularization over time. This allowed us to visually validate our CITS model by superimposing the average marginal estimates based on the interrupted time series model (which did not assume that time trends were linear) on the graphs based on the CITS model (which assumed that time trends were linear).

Second, we repeated the main analyses after excluding patients who were transferred out since these patients may not have been revascularized during the index admission but could have been revascularized following transfer. Third, we repeated the main analyses after first excluding states that provided Medicaid coverage similar to the ACA’s Medicaid expansion prior to 2014: Washington, D.C., Delaware, Massachusetts, New York, and Vermont [35].

Fourth, we used mixed-effects modeling (hospitals and states were specified as random effects) to examine the association between Medicaid expansion and disparities in revascularization. Note that in our main analyses, we specified hospitals as fixed effects because this approach allowed us to control for unmeasured hospital-level confounding.

Fifth, we performed a post-hoc analysis to examine the association between the ACA and disparities in revascularization in elderly patients (age 65 and over). We performed this post-hoc analysis to determine whether the reduction in revascularization disparity after the implementation of the ACA in non-elderly STEMI patients was also present in elderly patients. The study cohort for the post-hoc analysis consisted of 69,081 STEMIs (S1 Appendix Fig 1C).

Data management and statistical analyses were performed using STATA SE/MP Version 16.0 (Stata Corp, USA). All statistical tests were two-tailed and P values less than 0.05 were considered significant.

## Results

### Patient population

The analysis was based on hospitalizations for 68,610 STEMIs and 127,378 NSTEMIs in 175 hospitals in patients under the age of 65 (S1 Appendix Fig 1A and 1B). The percentage of black patients under 65 was 18.3% for STEMIs and 25.8% for NSTEMIs, with higher percentages of blacks in non-expansion states compared to expansion states (P<0.001) (S1 Appendix Tables 3 and 4). Most patients were male (76.1% and 66.7%, STEMI and NSTEMI, respectively). Thirteen percent of the STEMI patients and 14.2% of the NSTEMI patients had a prior PCI.

### Trends in insurance coverage

The percentage point decrease in the uninsured rate after Medicaid expansion was greater for expansion states versus non-expansion states for STEMIs (9.9% vs 6.4%, P < 0.001) and for NSTEMIs (9.6% vs 5.0%, P <0.001) (S1 Appendix Fig 2A and 2B). Uninsured rates were between three to four times higher in non-expansion states compared to expansion states after the ACA for STEMIs (19.2% vs 4.8%, P < 0.001) and NSTEMIs (18.2% vs 3.7%, P < 0.001) (S1 Appendix Fig 2A and 2B).

The percentage point decrease in the uninsured rate for STEMIs was greater for blacks in expansion states compared to white in expansion states (14% vs 9.2%, P < 0.001) but not in non-expansion states (6.1% vs 6.4%, P = 0.75) (S1 Appendix Fig 3). The percentage point decrease in the uninsured rate for NSTEMIs was greater for blacks in expansion states compared to white in expansion states (12.7% vs 8.8%, P < 0.001), but was not significantly different for blacks versus whites in non-expansion states (5.5% vs 5.0%, P = 0.46) (S1 Appendix Fig 4A and 4B).

### Association between the ACA and medicaid expansion and racial disparities in revascularization for STEMI

**Fig 1:**
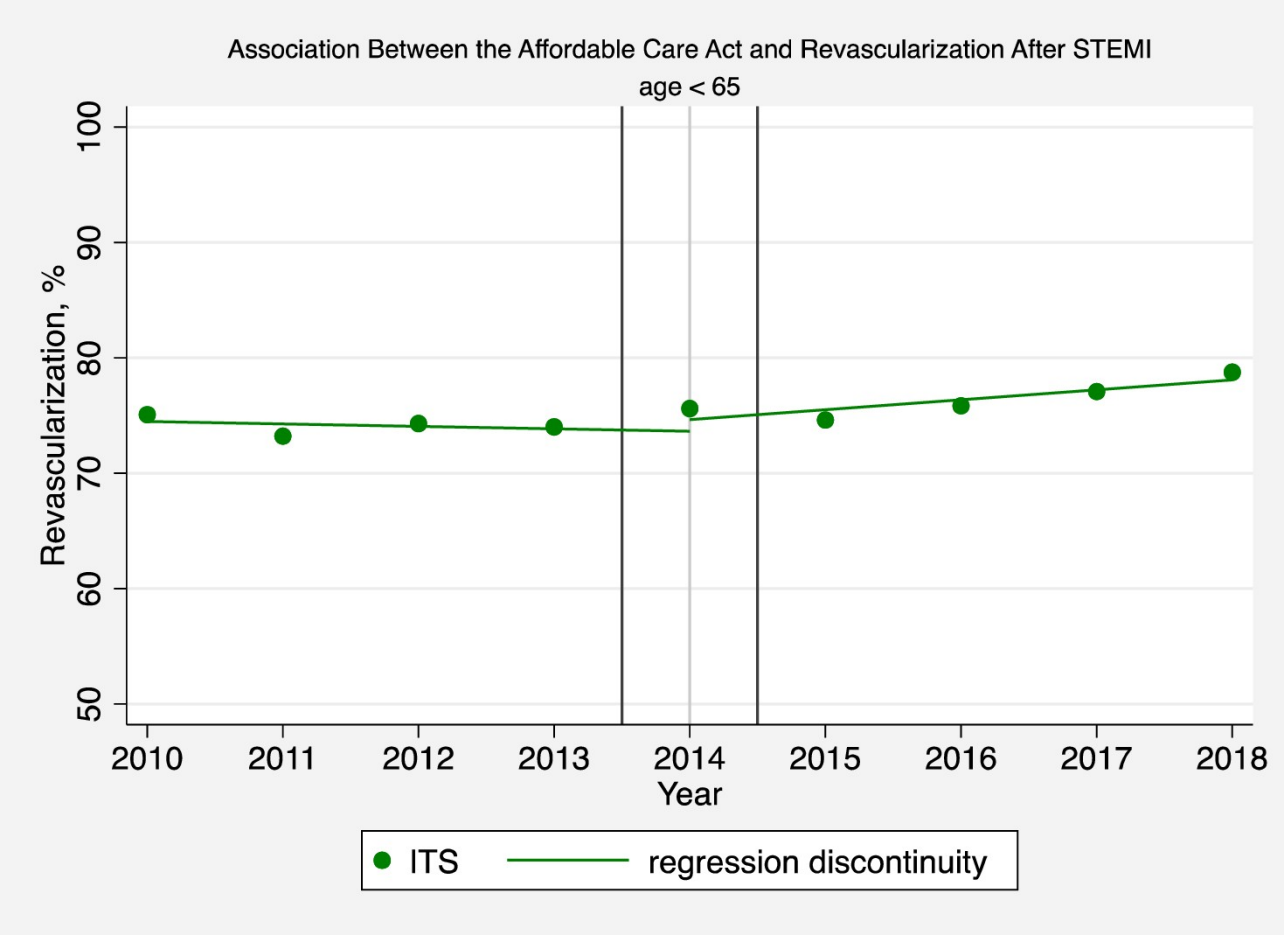
Association between the affordable care act and revascularization after STEMI (age < 65). The solid circles represent the yearly estimates for the revascularization rate in patients hospitalized with a STEMI based on an interrupted time series model after adjusting patient-level and hospital effects, without imposing restrictions on time trends and making no assumptions that time effects were different before or after 2014. The solid line is based instead on a regression discontinuity model which assumes that pre-intervention and post-intervention trends are linear in order to interpret changes in revascularization rates before and after the ACA.

**Fig 2:**
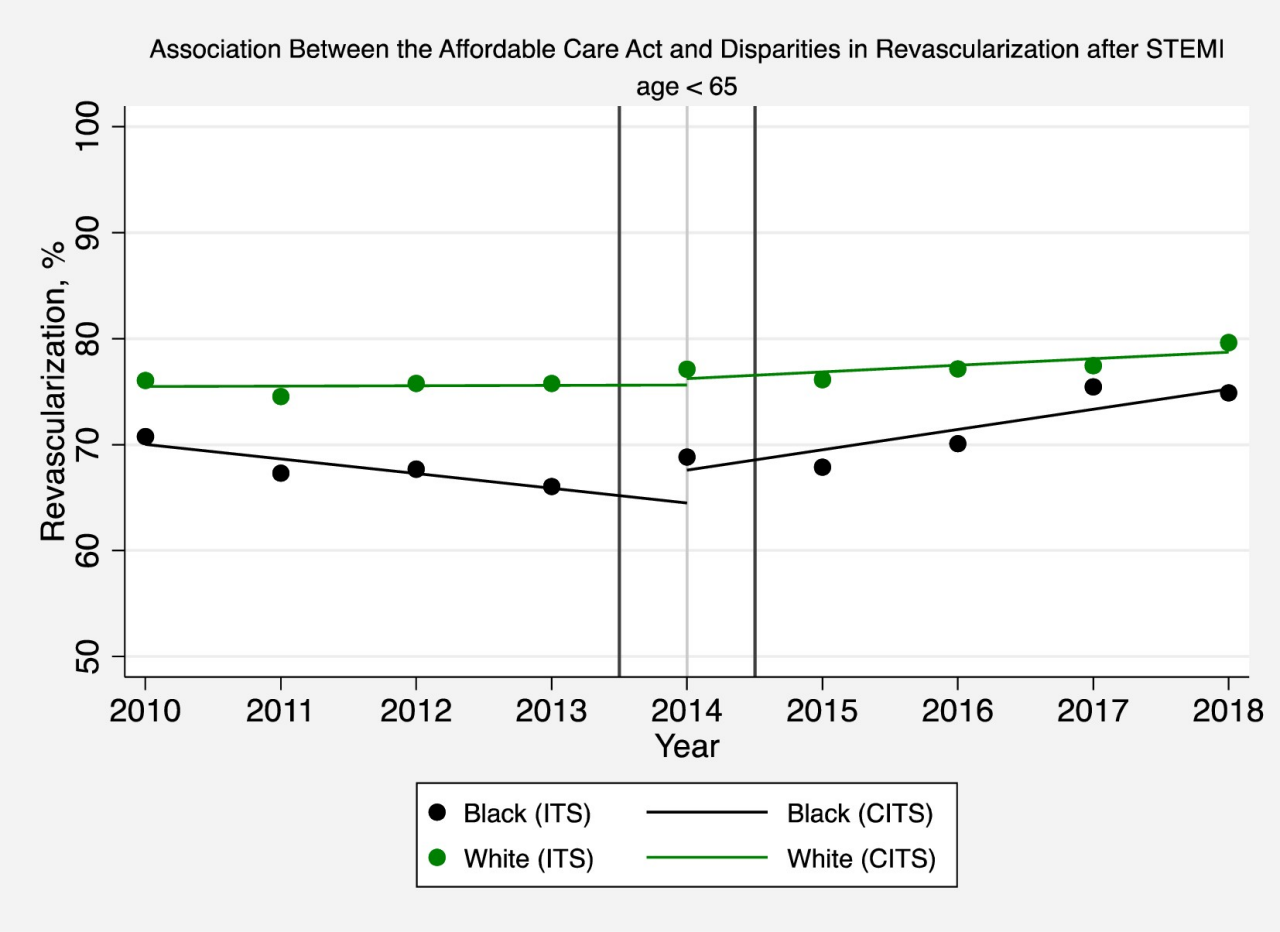
Association between the affordable care act and disparities in revascularization after STEMI (age < 65). The solid circles represent the yearly estimates for the revascularization rate in black and white patients hospitalized with a STEMI based on an interrupted time series (ITS) model after adjusting patient-level and hospital effects, without imposing restrictions on time trends and making no assumptions that time effects were different before or after 2014. The solid lines are based instead on a comparative interrupted time series (CITS) model which assumes that pre-intervention and post-intervention trends are linear in order to interpret changes in revascularization rates before and after Medicaid expansion.

For all patients younger than 65 (after controlling for patient characteristics, pre-ACA temporal trends, and hospital effects) implementation of the ACA was associated was associated with a 1.08 percentage point per year increase in the annual rate of revascularization (95% confidence interval [CI]: 0.45, 1.72; P = 0.001) (Fig 1 and S1 Appendix Table 5). However, white patients younger than 65 did not experience a significant increase in the rate of revascularization after the ACA was implemented (Fig 2 and S1 Appendix Table 5). But, differences in black versus white revascularization rates decreased by 2.77 percentage points per year (95% CI: 1.10, 4.44; P = 0.001) after the ACA was implemented (Fig 2 and S1 Appendix Table 5).

**Fig 3:**
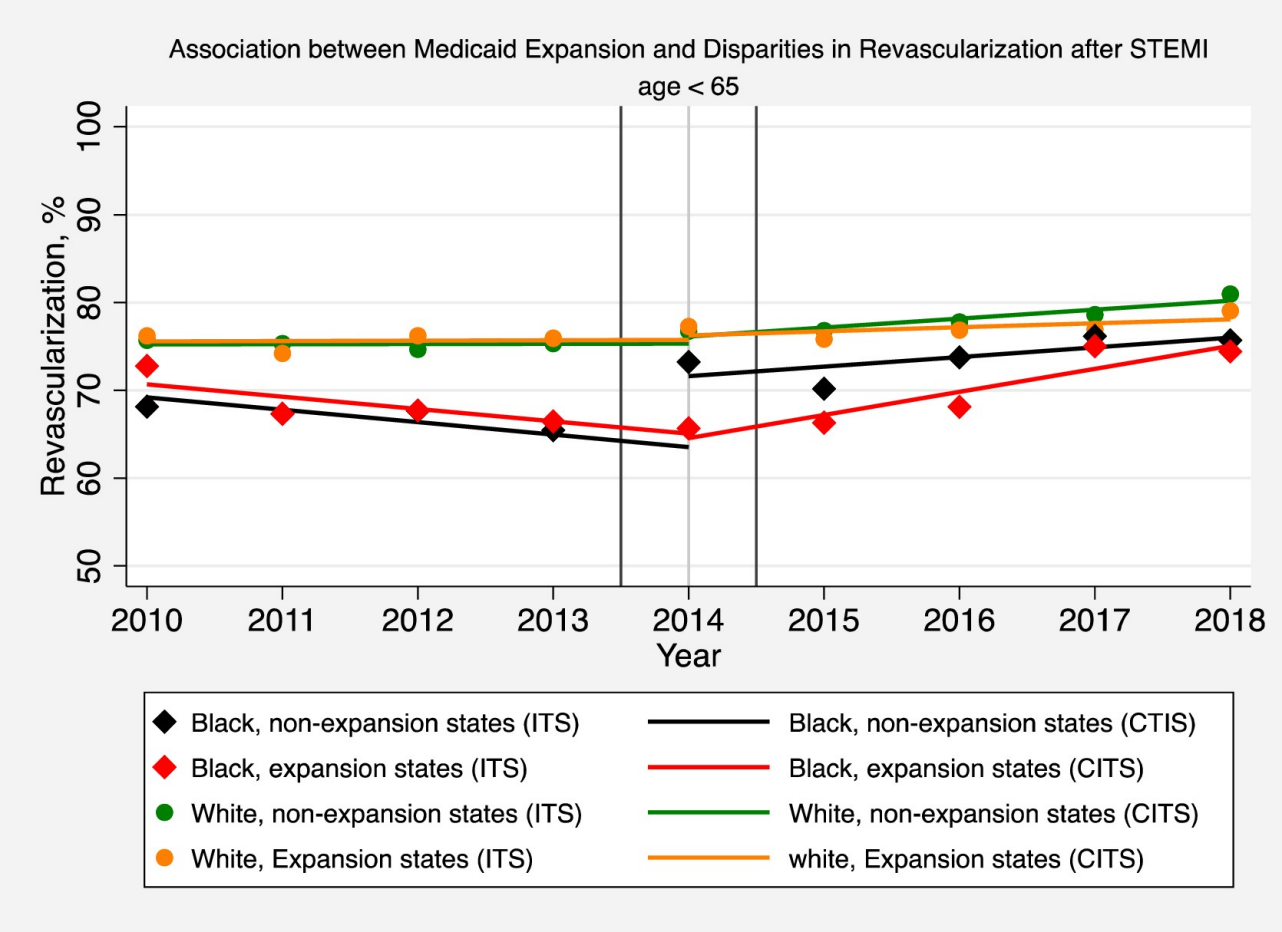
Association between medicaid expansion and disparities in revascularization after STEMI (age < 65). The solid symbols represent the yearly estimates for the revascularization rate in patients hospitalized with a STEMI in non-expansion states and expansion states, respectively, based on an interrupted time series (ITS) model after adjusting patient-level and hospital effects, without imposing restrictions on time trends and making no assumptions that time effects were different before or after 2014. The solid lines are based instead on a comparative interrupted time-series (CITS) model which assumes that pre-intervention and post-intervention trends are linear in order to interpret changes in revascularization rates before and after Medicaid expansion.

Medicaid expansion was not associated with annual increases in the rate of revascularization for white patients younger than 65 hospitalized with STEMIs (-0.58; 95% CI, -1.87, 0.71, P = 0.37) (Fig 3 and S1 Appendix Table 6). Differences in black versus white revascularization rates deceased by 2.09 percentage points per year (95% CI: 0.29, 3.88; P = 0.023) in expansion versus non-expansion states after adjusting for patient and hospital characteristics. Black patients in non-expansion states did not experience a significant increase in the annual rate of revascularization compared to whites in non-expansion states (1.52; 95% CI, -0.51, 3.55; P = 0.14). However, black patients in non-expansion states experienced a significant 7.24%-point increase in the revascularization rate in 2014 (intercept shift) compared to whites in non-expansion states (95% CI: 2.83, 11.66; P = < 0.001) (Fig 3 and S1 Appendix Table 6).

**Fig 4:**
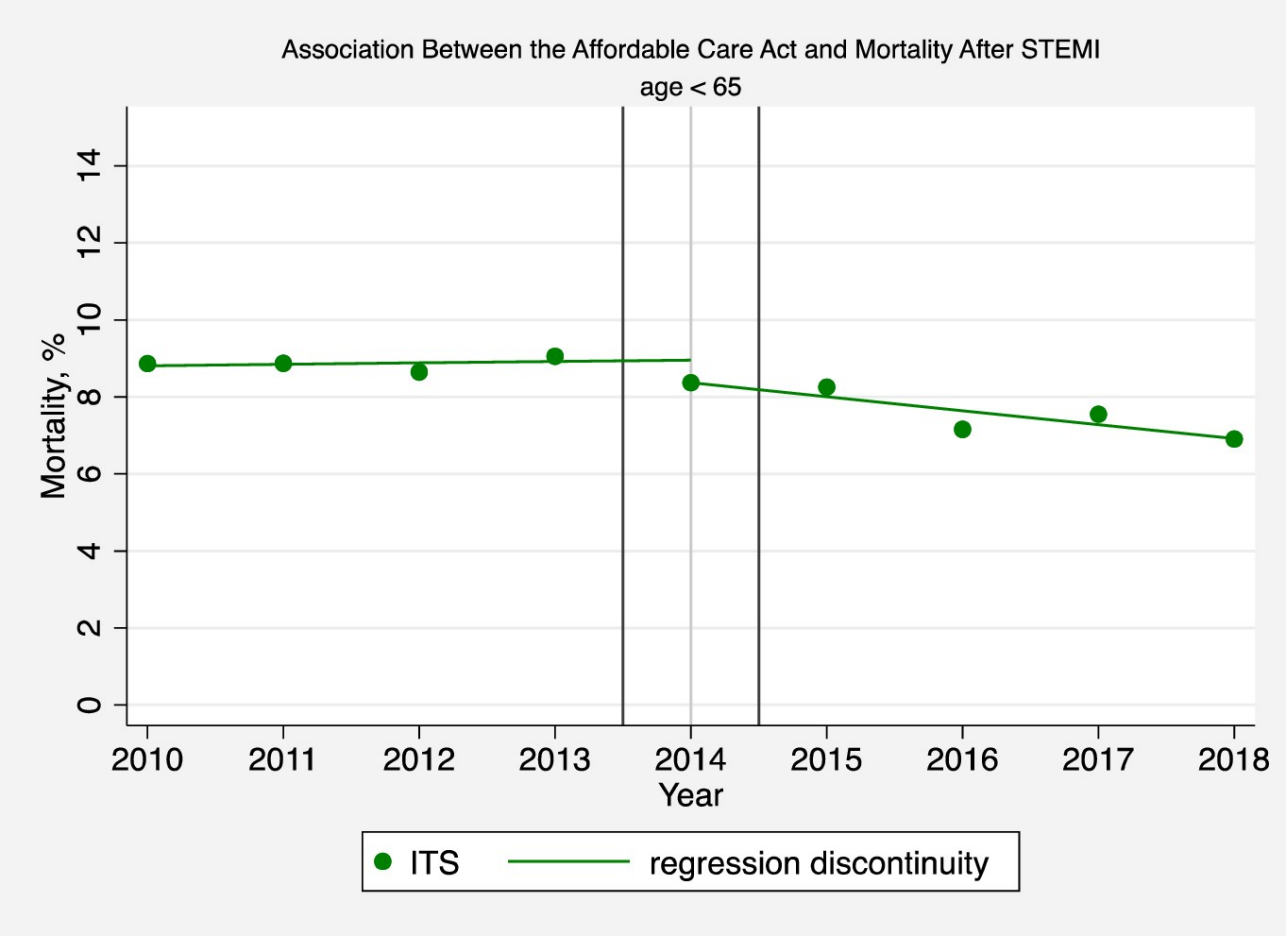
Association between the affordable care act and mortality after STEMI (age < 65). The solid circles represent the yearly estimates for the mortality rate in patients hospitalized with a STEMI based on an interrupted time series (ITS) model after adjusting patient-level and hospital effects, without imposing restrictions on time trends and making no assumptions that time effects were different before or after 2014. The solid line is based instead on a regression discontinuity model, and assumes that pre-intervention and post-intervention trends are linear in order to interpret changes in mortality rates before and after the ACA.

**Fig 5:**
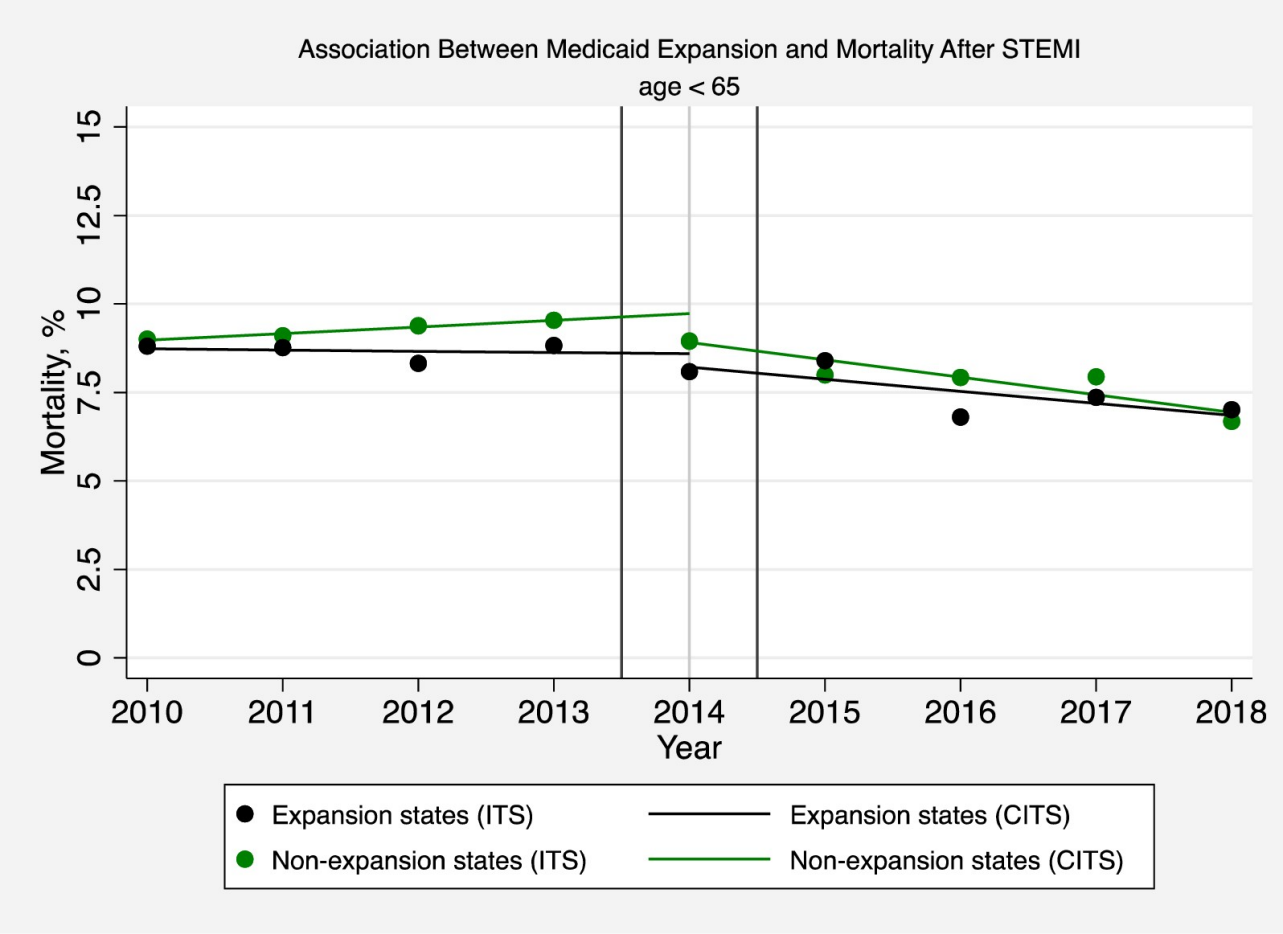
Association between medicaid expansion and mortality after STEMI (age < 65). The solid circles represent the yearly estimates for the mortality rate in patients hospitalized with a STEMI based on an interrupted time series (ITS) model after adjusting patient-level and hospital effects, without imposing restrictions on time trends and making no assumptions that time effects were different before or after 2014. The solid lines are based instead on a comparative interrupted time-series (CITS) model but assume that pre-intervention and post-intervention trends are linear in order to interpret changes in mortality rates before and after Medicaid expansion.

In secondary analyses, we found that the ACA was associated with annual decreases in the odds of STEMI in-hospital mortality in patients younger than 65 (adjusted odds ratio [AOR] 0.92; 95% CI, 0.87–0.98, P = 0.009) (Fig 4 and S1 Appendix Table 7). There was no change in STEMI mortality in expansion versus non-expansion states (AOR 1.07; 95% CI, 0.95–1.20, P = 0.27) (Fig 5 and S1 Appendix Table 7).

### Association between the ACA and medicaid expansion and racial disparities in revascularization for NSTEMI

For patients younger than 65 (and, after controlling for patient characteristics, pre-ACA temporal trends, and hospital effects), implementation of the ACA was associated with a 1.21 percentage point increase in revascularization rate in 2014 (i.e. intercept shift) (95% CI, 0.08, 2.35, P = 0.037), but was not associated with annual increases in the revascularization rate per year after 2014 (-0.25, 95% CI -0.79–0.30, P = 0.37) (S1 Appendix Fig 5 and S1 Appendix Table 5). Black and white patients younger than 65 did not experience significant changes in revascularization rates following the implementation of the ACA (S1 Appendix Fig 6 and S1 Appendix Table 5). Black and white patients younger than 65 living in expansion states did not experience significant changes in revascularization rates compared to blacks and whites in non-expansion states (S1 Appendix Fig 7 and S1 Appendix Table 6).

In secondary analyses, we found that the ACA was not associated with annual decreases in the odds of NSTEMI in-hospital mortality for patients younger than 65 (AOR 0.96; 95% CI, 0.91–1.02, P = 0.16) (S1 Appendix Fig 8 and S1 Appendix Table 7). Medicaid expansion was also not associated with changes in mortality in patients younger than 65 (AOR 0.95; 95% CI, 0.89,1.02, P = 0.18) (S1 Appendix Fig 9 and S1 Appendix Table 7).

### Results of sensitivity analyses

First, we estimated separate interrupted time series (ITS) models for each of the above analyses to verify that the assumption of linearity of the pre and post-intervention trends in the CITS models were reasonable. The average marginal effects estimated using these ITS models were superimposed on the average marginal effects estimated using the CITS models. Visual examination of all of the figures revealed that the assumption of linearity of pre and post trends in the CITS models were justified.

Second, the results of the sensitivity analyses in which we excluded states that expanded Medicaid coverage before 2014 (17.5% of the cases were excluded) were very similar to the results from the baseline analyses (S1 Appendix Figures 10–13).

Third, the results of the sensitivity analysis in which we used mixed effects models were also very similar to those obtained using fixed-effects modeling, although the confidence intervals were slightly more conservative (wider) with fixed effects modeling (S1 Appendix Table 8). Finally, the results of sensitivity analyses in which we excluded 2.61% of STEMI records and 3.81% of NSTEMI patients transferred out were nearly unchanged compared to our main analyses (results not shown).

### Results of post-hoc analysis

For patients 65 and older, the ACA was associated with a 2.25 percentage point annual increase (95% CI: 1.66, 2.84; P < 0.001) in the revascularization rate after controlling for patient characteristics, pre-ACA temporal trends, and hospital effects (S1 Appendix Fig 14 and S1 Appendix Table 9). Blacks and white experienced similar annual increases in the revascularization rate after the ACA was implemented (P = 0.261) (S1 Appendix Fig 15 S1 Appendix Table 9).

## Discussion

Medicaid expansion was associated with a greater reduction in the number of uninsured black patients compared to uninsured white patients. Despite narrowing the insurance gap between black and white patients, Medicaid expansion was not associated with decreased racial disparities in revascularization for patients hospitalized with a STEMI or NSTEMI. Instead, we found that black STEMI patients experienced significant increases in revascularization rates in both expansion and non-expansions states compared to white patients.

It is unknown whether the increase in revascularization rates in black STEMI patients in expansion and non-expansion states after 2014 was due to the implementation of the ACA or due to the adoption of the updated 2014 AHA/ACC guidelines that recommended early invasive therapy for STEMIs.

On the one hand, we found that both black and white STEMI elderly patients experienced similar increases in revascularization rates after 2014 which is consistent with adoption of the updated 2014 guidelines. On the other hand, while non-elderly black STEMI patients experienced increased revascularization rates after 2014, non-elderly white STEMI patients did not. If the updated guidelines were responsible for changes in STEMI revascularization after 2014, one would expect to see increased revascularization rates in both non-elderly whites and non-elderly blacks. Furthermore, since the 2007 guidelines recommended early invasive therapy for STEMIs, the updated 2014 guidelines would not have been expected to have as much impact on clinical practice as a new set of guidelines. It is plausible that other parts of the ACA besides Medicaid expansion, such as the elimination of exclusions for pre-existing conditions and the provision of income-based subsidies, may have helped to reduce disparities in vulnerable patient populations. It is also possible that cardiologists, regardless of whether they treat patients in expansion or non-expansion states, may have responded to the ACA by expanding the use of lifesaving invasive therapies in STEMI patients, irrespective of insurance status. Nonetheless, decreases in revascularization disparities in non-elderly STEMI patients after 2014 may simply reflect efforts by cardiologists and surgeons to increase the use of indicated revascularization therapies in blacks and other under-served populations in light of the renewed emphasis on the use of invasive therapies for STEMI patients recommended in the updated 2014 guidelines.

To our knowledge, ours is the first study to show that Medicaid expansion was not associated with decreases in racial disparities in revascularization. Using data from the National Cardiovascular Get With the Guidelines Registry, Wadhera et. al. recently reported that Medicaid expansion was associated with less of a decrease in the use of PCI in low-income patients admitted with STEMI, and no change in PCI for low-income patients admitted with NSTEMI [35]. Although Wadhera et. al. did not explicitly examine the effect of Medicaid expansion on racial disparities, there is some overlap between our study and Wadhera et al. since blacks are twice as likely to live in poverty compared to whites [36]. However, our hospital sample may not be comparable to the hospitals included in the Get With the Guidelines Registry, which is a voluntary association of hospitals dedicated to improving the care of STEMI patients. In particular, the rate of primary PCI in STEMI patients in the Get With the Guidelines Registry is over 95%, whereas the baseline rate in our sample is less than 80% for STEMIs, thus providing greater opportunities for improvement in the hospitals included in our analyses [35].

Using data from the Centers for Disease Control, Khatana recently reported that counties that expanded Medicaid access experienced smaller increases in cardiovascular death rates among non-elderly adults compared to counties that did not expand Medicaid access [15]. We found instead that in-hospital mortality rates for STEMIs, but not NSTEMIs, decreased to the same extent in expansion and non-expansion states after the implementation of the ACA. Of note, we did not investigate the effects of Medicaid expansion on disparities in mortality because we found that blacks and whites had similar in-hospital mortality for STEMIs while blacks had slightly lower in-hospital mortality for NSTEMIs. It is possible that lower black in-hospital NSTEMI mortality rates reflect higher early in-hospital mortality from the greater use of invasive therapies in whites versus blacks. In-hospital mortality, however, does not reflect the longer-term benefits of revascularization that occur after 12 months or more, which may contribute to well-documented disparities in long-term cardiovascular outcomes. Additional studies are necessary to determine whether increases in the utilization of revascularization therapies in black non-elderly adults hospitalized with STEMIs after 2014 have led to reductions in disparities in long-term cardiovascular outcomes.

### Limitations

There are several limitations to this study. First, because our study is observational in nature and is based on administrative data, it is possible that our findings are confounded by unobserved differences between patients in expansion and non-expansion states. However, our use of comparative interrupted time series analysis is a more robust approach to controlling for differences in pre-intervention time trends than more conventional difference-in-difference estimators typically used in this type of analysis. Second, our study is not population-based, and is therefore not necessarily generalizable outside of this large group of academic medical centers and their affiliated hospitals. Third, we were not able to include revascularizations that occurred after the initial hospitalization. This may have resulted in under-count of the number of revascularizations, particularly in the NSTEMI patients where the decision to perform revascularization may have been delayed. A priori, it is unlikely that this would have had a different effect on blacks versus whites.

We believe that our findings are relatively robust. We conducted multiple sensitivity analyses, including the use of interrupted time series models which do not impose linear restrictions on rates of revascularization over time. We also used mixed-effects modeling in which we specified hospitals as a random effect instead of as fixed effects. Finally, we repeated our main analyses after first excluding states that provided Medicaid coverage similar to the ACA’s Medicaid expansion prior to 2014. The results of these sensitivity analyses were consistent with the results of our main analyses.

## Conclusions

In summary, we found that Medicaid expansion was associated with greater reductions in the number of uninsured blacks compared to uninsured whites. Medicaid expansion was not associated, however, with reduction in revascularization disparities between black and white patients admitted with acute myocardial infarctions.

## Supporting information

S1 Appendix (DOCX)
