# Supplementary material for: Impact of medicaid expansion on disparities in revascularization in patients hospitalized with acute myocardial infarction
Source: PLoS One. 2020 Dec 23;15(12):e0243385. doi: 10.1371/journal.pone.0243385 (PMC7757880; doi:10.1371/journal.pone.0243385)
Supplement: S1 Appendix — (DOCX) [file pone.0243385.s001.docx]

**SUPPLEMENTARY APPENDIX TABLES**

**Appendix Table 1.** Procedure codes used to define patient populations and procedures.

**Appendix Table 2.** Definition of Expansion and Non-expansion states.

**Appendix Table 3.** Patient demographics for STEMI.

**Appendix Table 4.** Patient demographics for NSTEMI.

**Appendix Table 5.** Association between the ACA and disparities in Revascularization in STEMI and NSTEMI patients (age less than 65).

**Appendix Table 6.** Association between Medicaid expansion and Disparities in Revascularization in STEMI and NSTEMI patients (age less than 65).

**Appendix Table 7.** Association between the ACA and Medicaid Expansion and STEMI and NSTEMI mortality (age less than 65).

**Appendix Table 8.** Association between Medicaid expansion and Disparities in Revascularization in STEMI and NSTEMI patients: comparison of fixed-effect versus random-effect model (age less than 65).

**Appendix Table 9.** Association between the ACA and disparities in Revascularization in STEMI and NSTEMI patients (age >= 65).

**Appendix Table 10.** Association between Medicaid expansion and Disparities in Revascularization in STEMI and NSTEMI patients (age >= 65).

**APPENDIX FIGURES**

**Appendix Figure 1a.** Flow diagram for creation of analytic data set for STEMI admissions (age < 65).

**Appendix Figure 1b.** Flow diagram for creation of analytic data set for NSTEMI admissions (age < 65).

**Appendix Figure 1c.** Flow diagram for creation of analytic data set for STEMI admissions (age > 65).

**Appendix Figure 2.** Changes in Insurance after Medicaid Expansion in STEMI and NSTEMI patients.

**Appendix Figure 3.** Changes in Insurance after Medicaid Expansion in STEMI patients stratified by race.

**Appendix Figure 4.** Changes in Insurance after Medicaid Expansion in NSTEMI patients stratified by race.

**Appendix Figure 5.** Association between the ACA and Revascularization after NSTEMI (age < 65).

**Appendix Figure 6.** Association between the ACA and Disparities in Revascularization after NSTEMI (age < 65).

**Appendix Figure 7.** Association between the Medicaid Expansion and Disparities in Revascularization after NSTEMI (age < 65).

**Appendix Figure 8.** Association between the ACA and Mortality after NSTEMI (age < 65).

**Appendix Figure 9.** Association between Medicaid Expansion and Mortality after NSTEMI (age < 65).

**Appendix Figure 10.** Association between the ACA and Disparities in Revascularization after STEMI (age < 65)(excluding states that expanded Medicaid access prior to 2014).

**Appendix Figure 11.** Association between Medicaid expansion and Disparities in Revascularization after STEMI (age < 65)(excluding states that expanded Medicaid access prior to 2014).

**Appendix Figure 12.** Association between the ACA and Disparities in Revascularization after NSTEMI (age < 65)(excluding states that expanded Medicaid access prior to 2014).

**Appendix Figure 13.** Association between Medicaid expansion and Disparities in Revascularization after NSTEMI (age < 65).(excluding states that expanded Medicaid access prior to 2014).

**Appendix Figure 14.** Association between the ACA and revascularization after STEMI (age >= 65).

**Appendix Figure 15.** Association between the ACA and Disparities in Revascularization after STEMI (age >= 65)


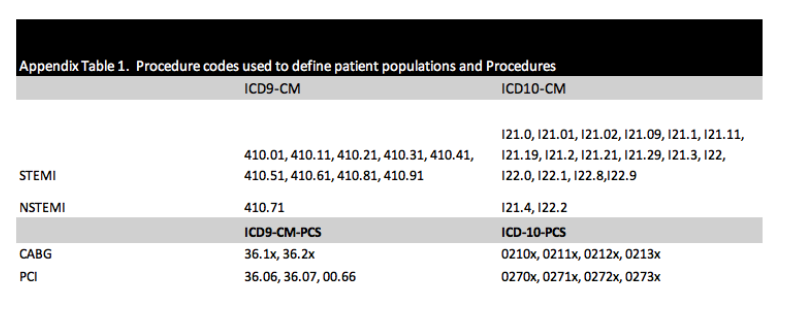


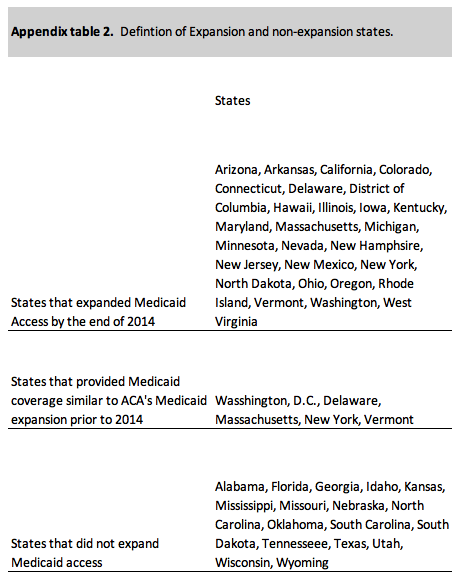


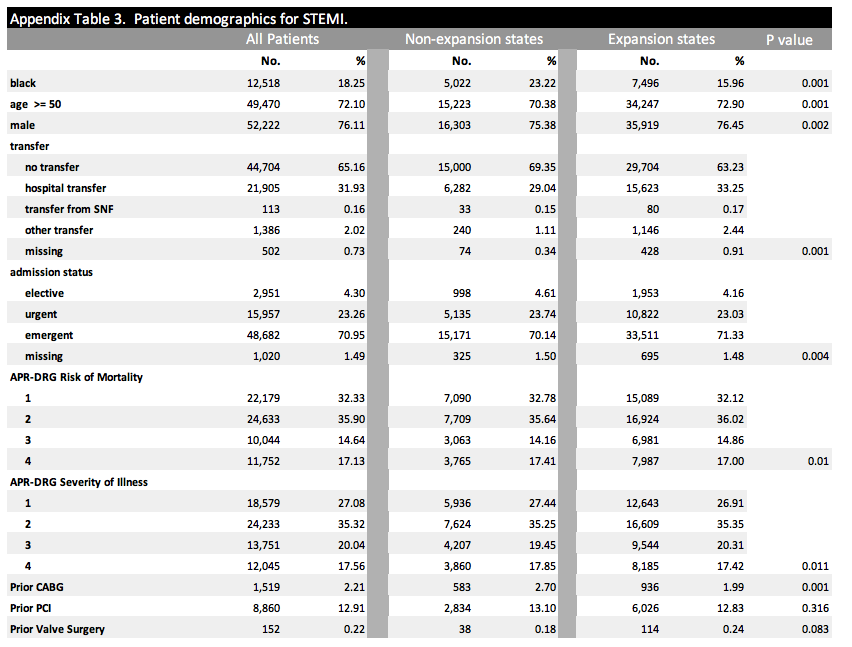

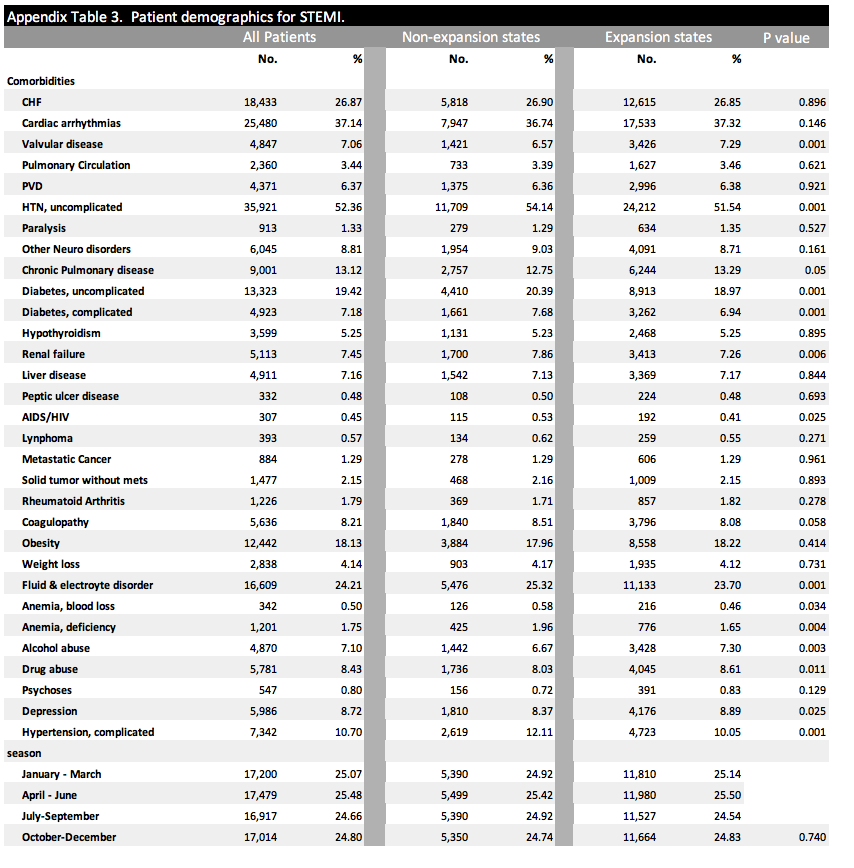


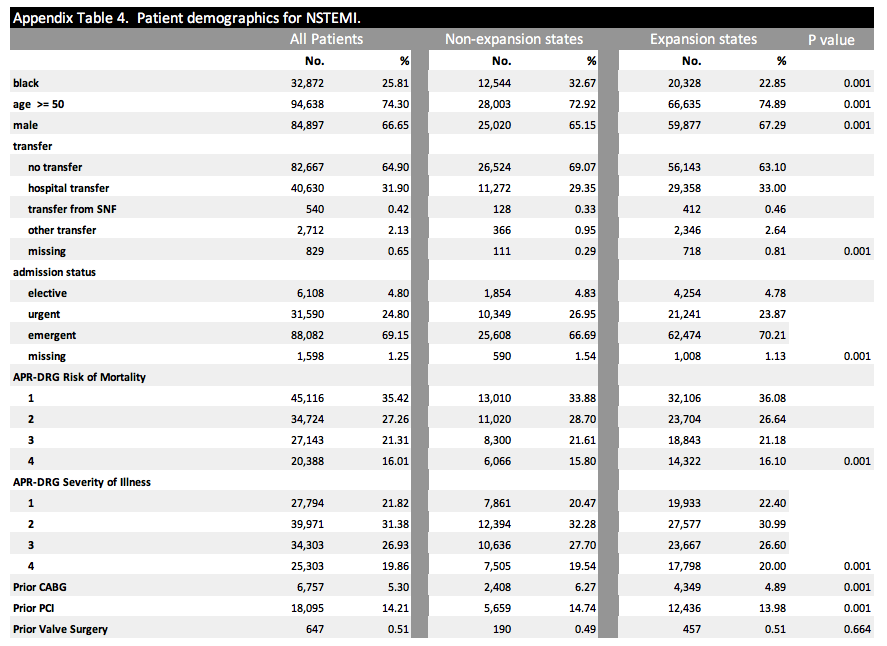


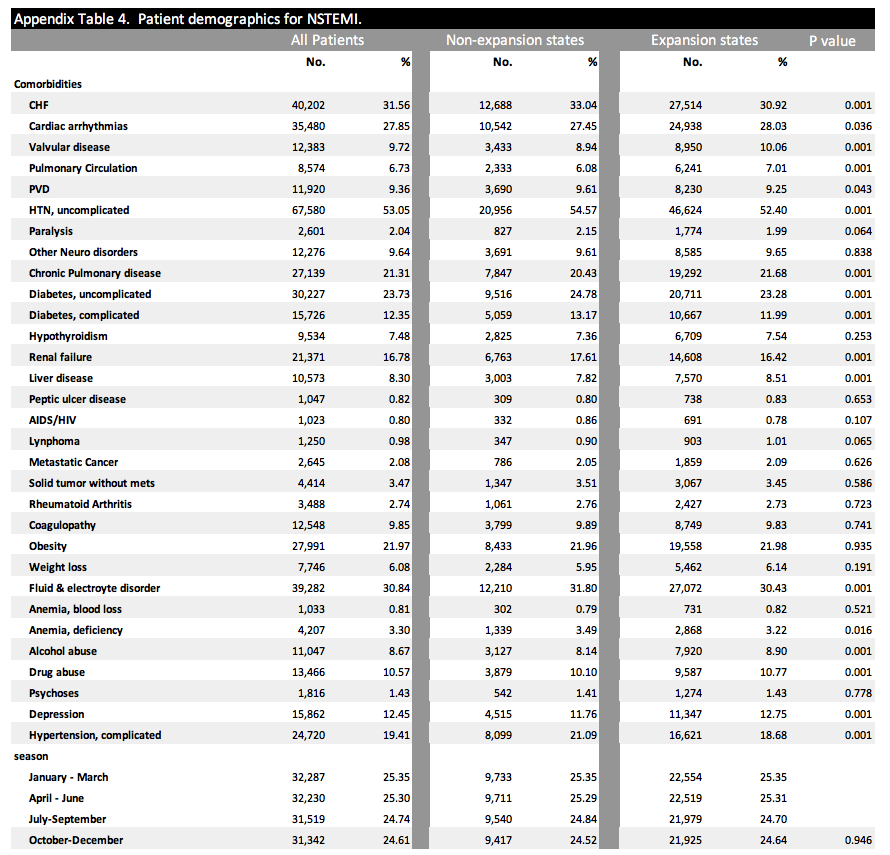


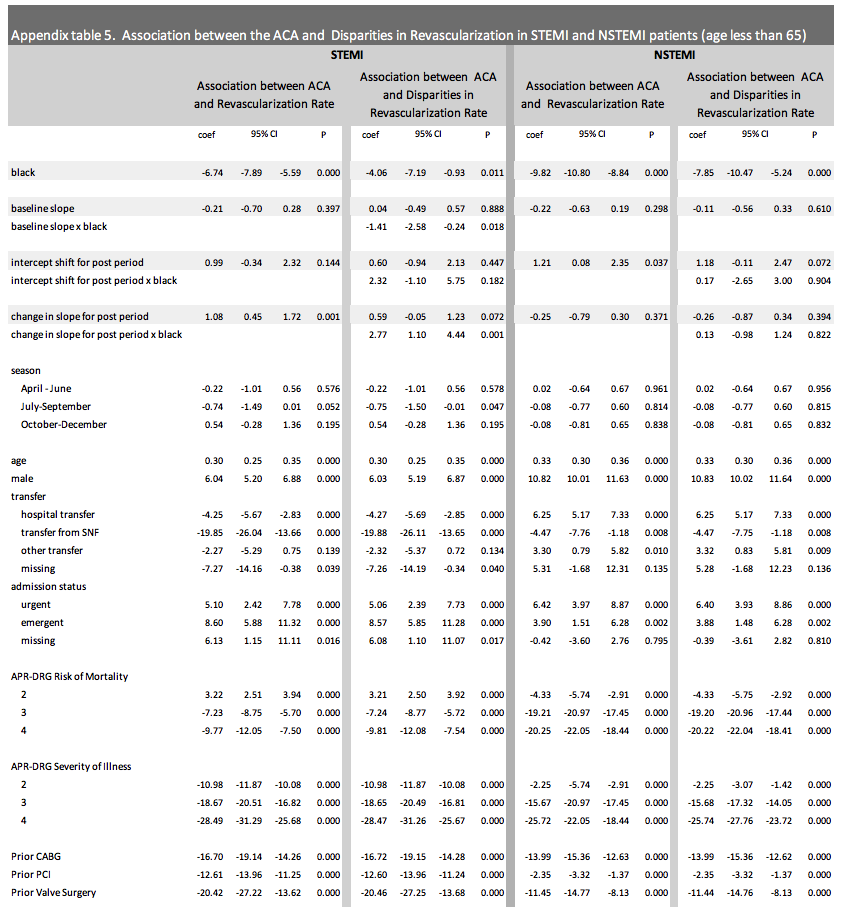


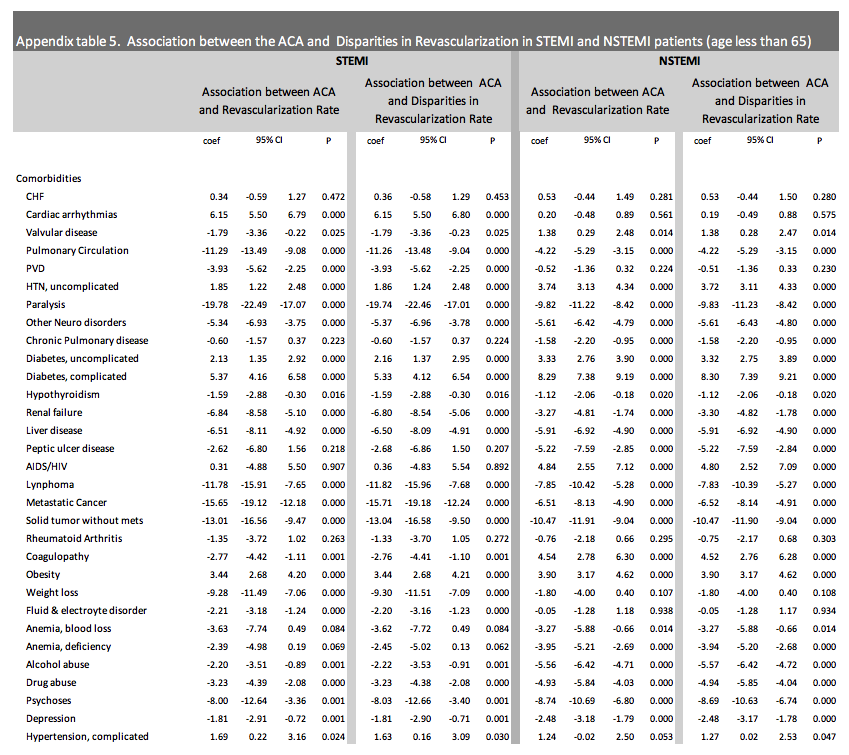


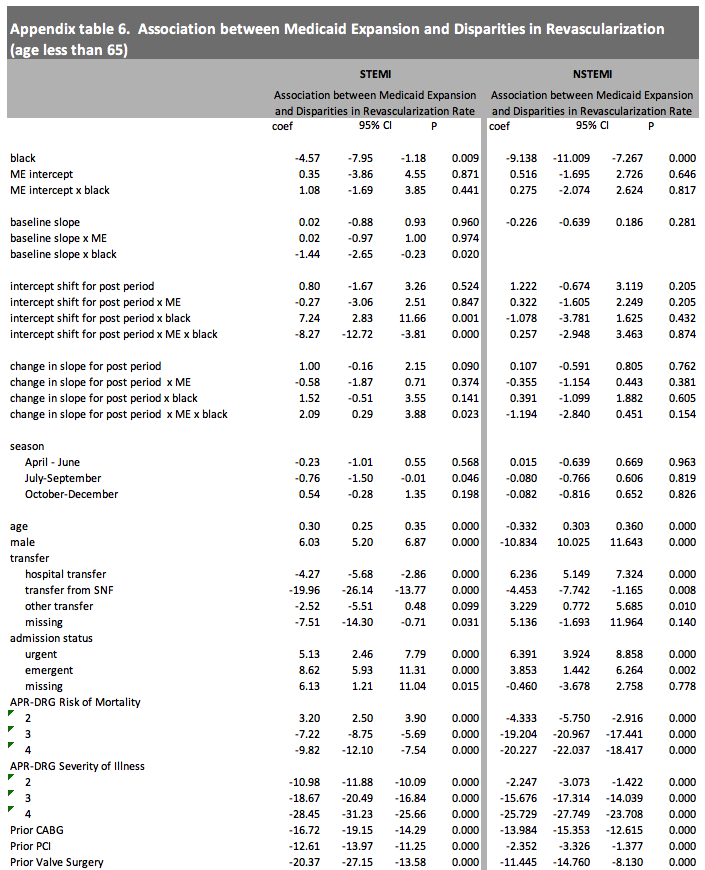


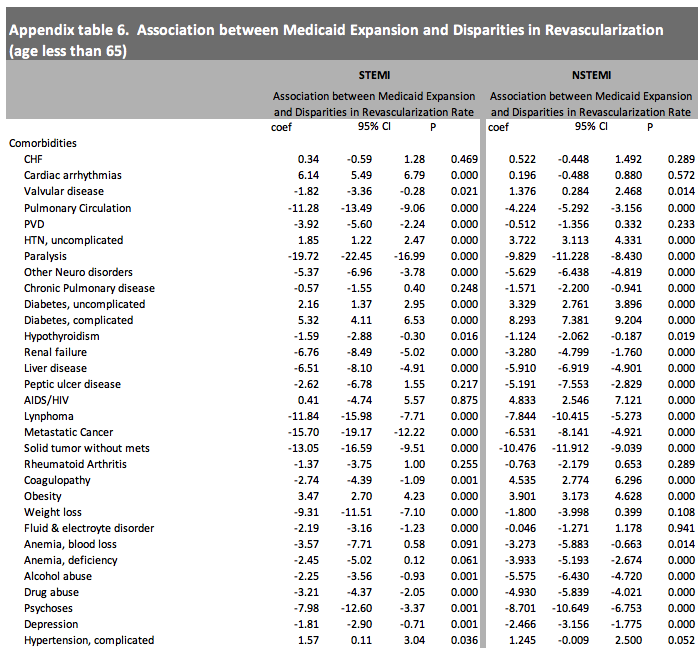


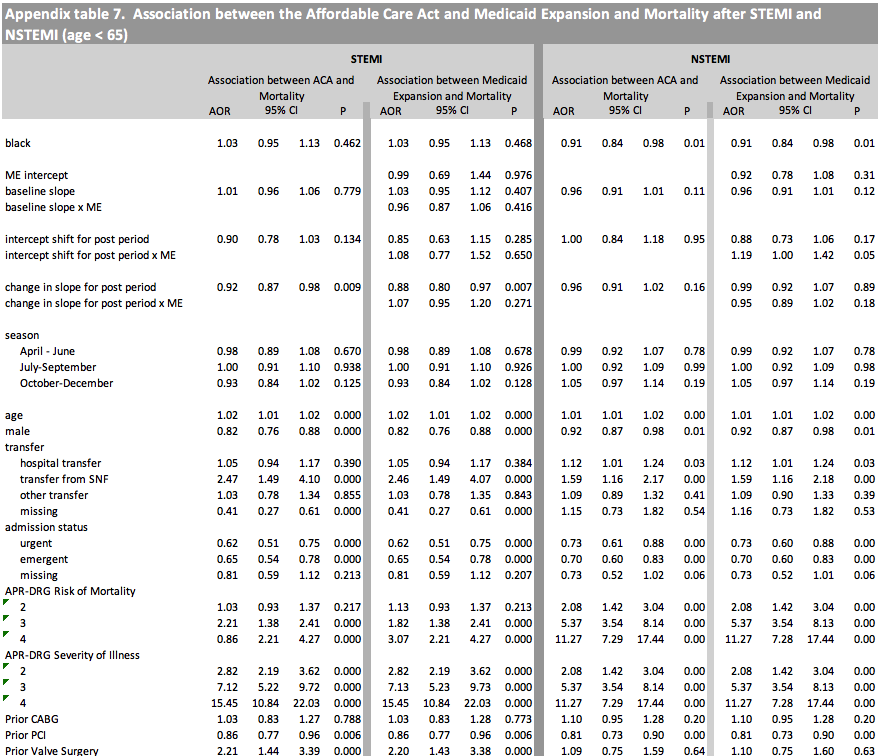


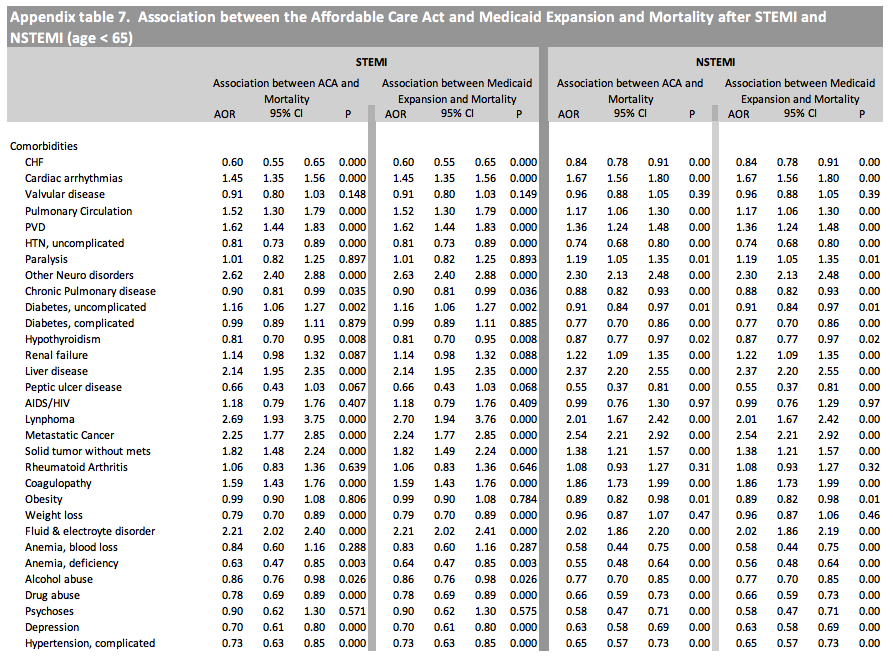


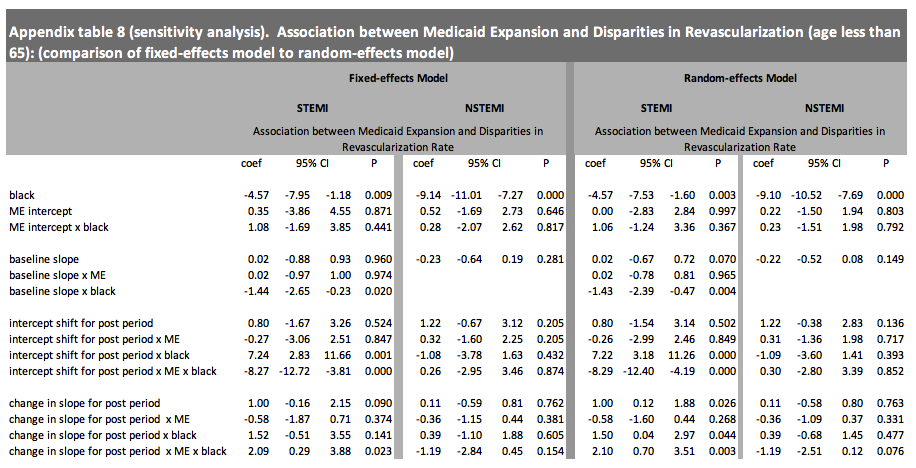


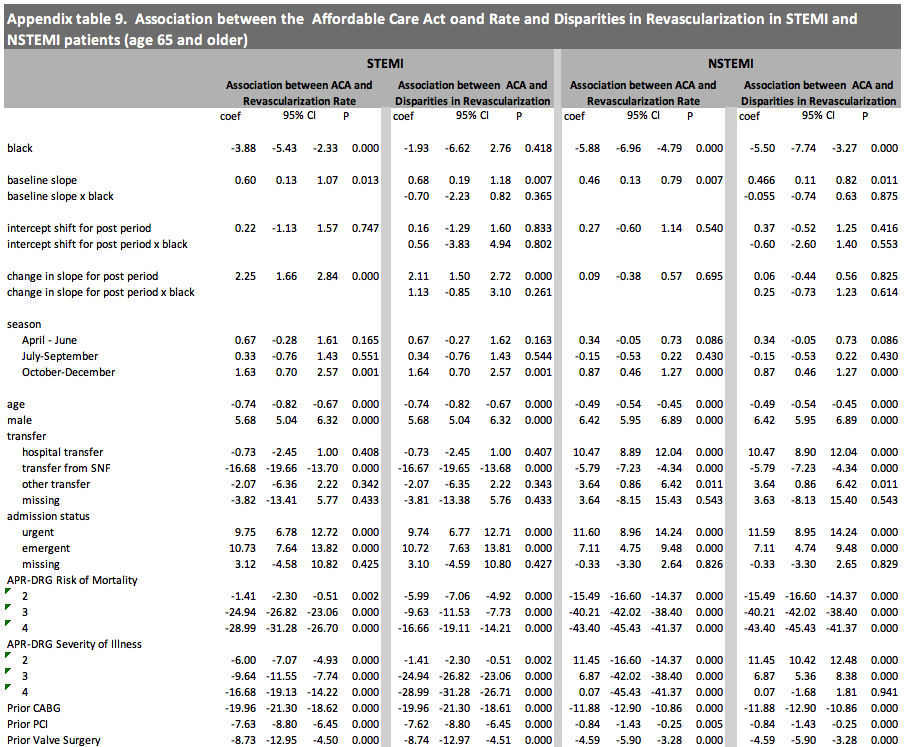


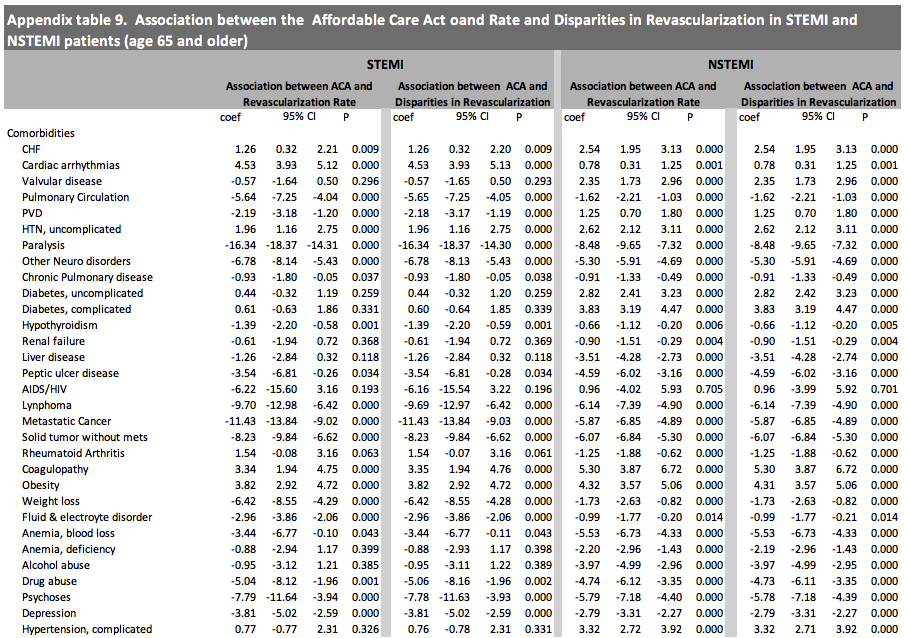


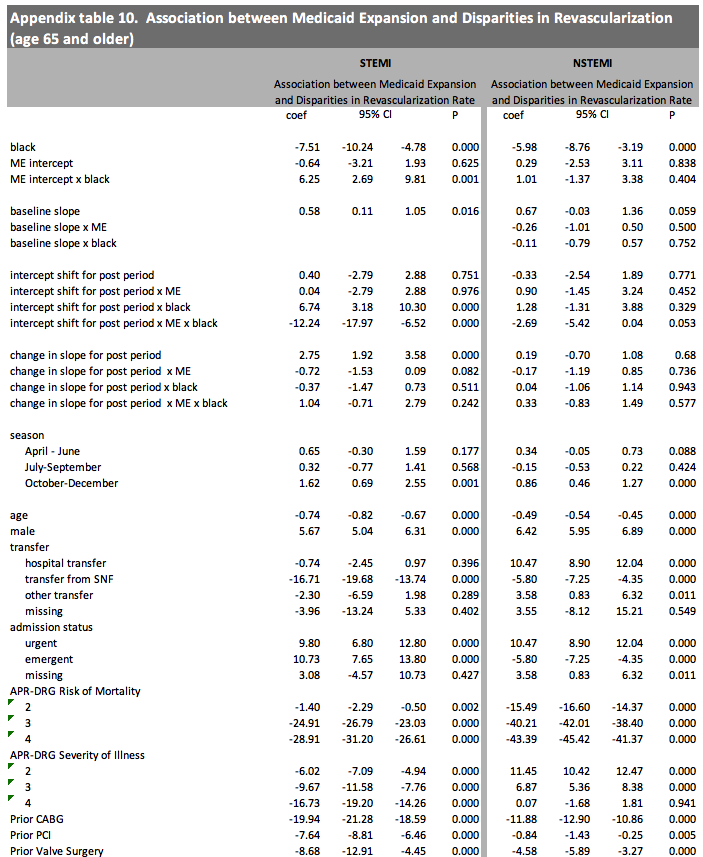


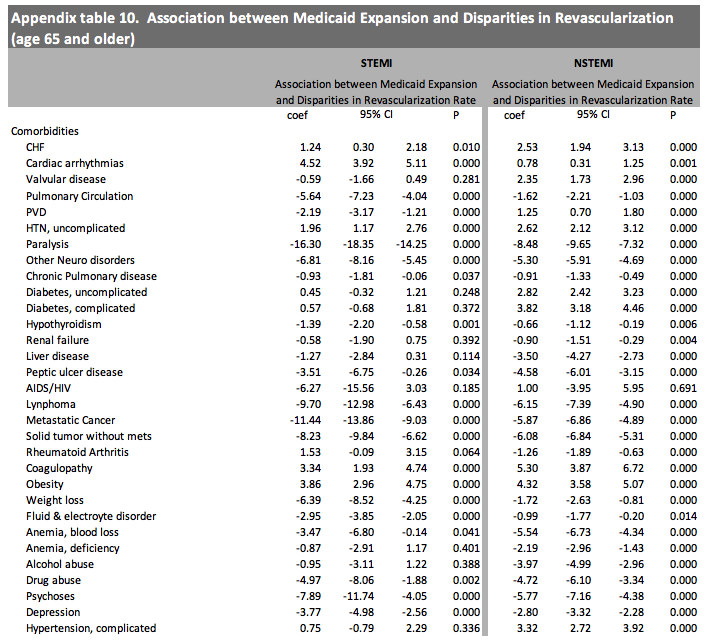


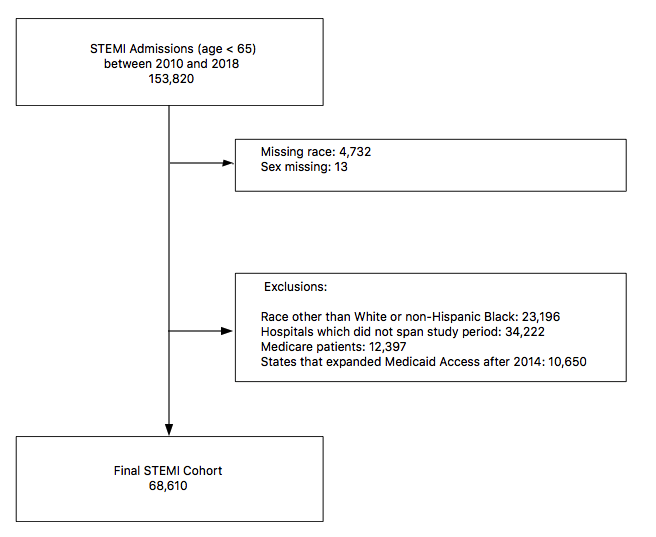


**Appendix Figure 1a.** Flow diagram for creation of analytic data set for STEMI admissions (age < 65).


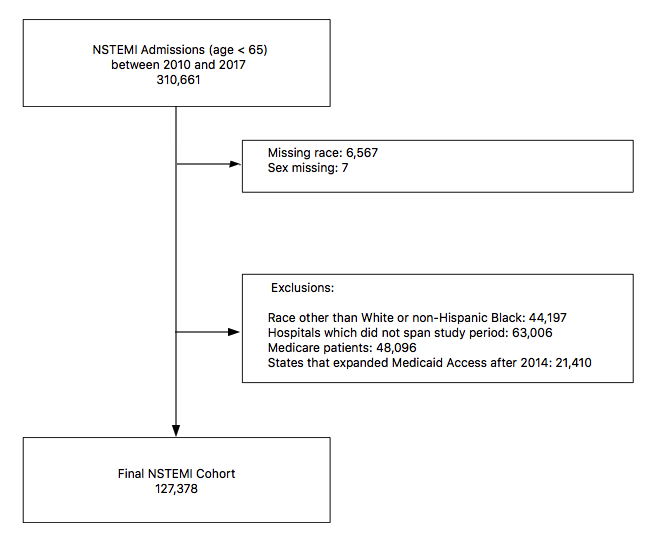


**Appendix Figure 1b.** Flow diagram for creation of analytic data set for NSTEMI admissions (age < 65).


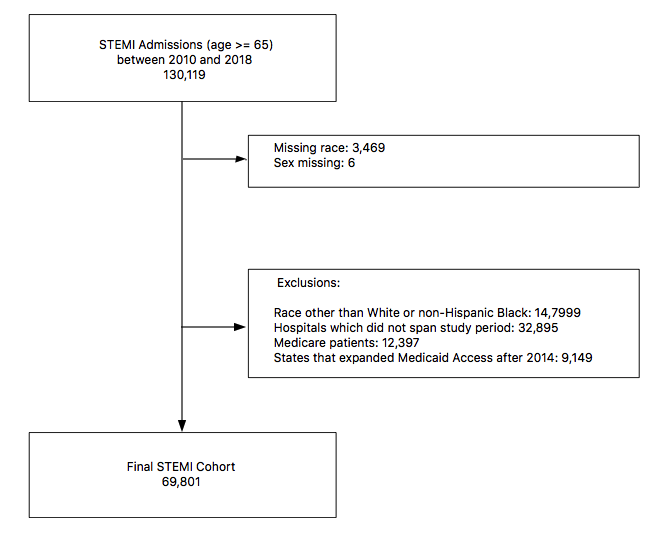


**Appendix Figure 1c.** Flow diagram for creation of analytic data set for STEMI admissions (age 65 and older).

**Figures 2a and 2b.** Proportion of patients hospitalized with STEMI (figure 7a) and NSTEMI (figure 7b) with Medicaid, private or other insurance versus no insurance. P < 0.001 for difference between expansion and non-expansion states in changes in insurance rates before and after the ACA.

**Appendix Figures 3a and 3b**. Proportion of black (figure 2a) and white (figure 2b) hospitalized with STEMI with Medicaid, private or other insurance versus no insurance. P < 0.001 for difference between blacks and whites in expansion versus non-expansion states in changes in uninsurance rates before and after the ACA.

**Appendix Figures 4a and 4b**. Proportion of black (figure 2a) and white (figure 2b) hospitalized with NSTEMI with Medicaid, private or other insurance versus no insurance. P < 0.001 for difference between blacks and whites in expansion versus non-expansion states in changes in uninsurance rates before and after the ACA.

**Appendix Figure 5.** The solid circles represent the yearly estimates for the revascularization rate in patients hospitalized with a NSTEMI based on an interrupted time series (ITS) model after adjusting patient-level and hospital effects, without imposing restrictions on time trends and making no assumptions that time effects were different before or after 2014. The solid lines are based instead on a regression discontinuity model which assumes that pre-intervention and post-intervention trends are linear in order to interpret changes in revascularization rates before and after the ACA.

**Appendix Figure 6.** The solid circles represent the yearly estimates for the revascularization rate in black and white patients hospitalized with a STEMI based on an interrupted time series (ITS) model after adjusting patient-level and hospital effects, without imposing restrictions on time trends and making no assumptions that time effects were different before or after 2014. The solid lines are based instead on a comparative interrupted time series (CITS) model which assumes that pre-intervention and post-intervention trends are linear in order to interpret changes in revascularization rates before and after Medicaid expansion.

**Appendix Figure 7.** The solid symbols represent the yearly estimates for the revascularization rate in patients hospitalized with an NSTEMI in non-expansion states and expansion states based on an interrupted time series (ITS) model after adjusting patient-level and hospital effects, without imposing restrictions on time trends and making no assumptions that time effects were different before or after 2014. The solid lines are based instead on a comparative interrupted time-series (CITS) model which assumes that pre-intervention and post-intervention trends are linear in order to interpret changes in revascularization rates before and after Medicaid expansion.

**Appendix Figure 8.** The solid circles represent the yearly estimates for the mortality rate in patients hospitalized with an NSTEMI based on an interrupted time series (ITS) model after adjusting patient-level and hospital effects, without imposing restrictions on time trends and making no assumptions that time effects were different before or after 2014. The solid line is based instead on a comparative interrupted time-series (CITS) model, and assumes that pre-intervention and post-intervention trends are linear in order to interpret changes in mortality rates before and after the ACA.

**Appendix Figure 9.** The solid circles represent the yearly estimates for the mortality rate in patients hospitalized with an NSTEMI based on an interrupted time series (ITS) model after adjusting patient-level and hospital effects, without imposing restrictions on time trends and making no assumptions that time effects were different before or after 2014. The solid lines are based instead on a comparative interrupted time-series (CITS) model but assume that pre-intervention and post-intervention trends are linear in order to interpret changes in mortality rates before and after Medicaid expansion.

**Appendix Figure 10.** The solid circles represent the yearly estimates for the revascularization rate in black and white patients hospitalized with a STEMI based on an interrupted time series (ITS) model after adjusting patient-level and hospital effects, without imposing restrictions on time trends and making no assumptions that time effects were different before or after 2014. The solid lines are based instead on a comparative interrupted time series (CITS) model which assumes that pre-intervention and post-intervention trends are linear in order to interpret changes in revascularization rates before and after Medicaid expansion.

This sensitivity analysis was conducted after excluding patients from Washington, D.C., Delaware, Massachusetts, New York, and Vermont.

**Appendix Figure 11.** The solid symbols represent the yearly estimates for the revascularization rate in patients hospitalized with a STEMI in non-expansion states and expansion states, respectively, based on an interrupted time series (ITS) model after adjusting patient-level and hospital effects, without imposing restrictions on time trends and making no assumptions that time effects were different before or after 2014. The solid lines are based instead on a comparative interrupted time-series (CITS) model which assumes that pre-intervention and post-intervention trends are linear in order to interpret changes in revascularization rates before and after Medicaid expansion.

This sensitivity analysis was conducted after excluding patients from Washington, D.C., Delaware, Massachusetts, New York, and Vermont.

**Appendix Figure 12.** The solid circles represent the yearly estimates for the revascularization rate in black and white patients hospitalized with a STEMI based on an interrupted time series (ITS) model after adjusting patient-level and hospital effects, without imposing restrictions on time trends and making no assumptions that time effects were different before or after 2014. The solid lines are based instead on a comparative interrupted time series (CITS) model which assumes that pre-intervention and post-intervention trends are linear in order to interpret changes in revascularization rates before and after Medicaid expansion.

This sensitivity analysis was conducted after excluding patients from Washington, D.C., Delaware, Massachusetts, New York, and Vermont

**Appendix Figure 13.** The solid symbols represent the yearly estimates for the revascularization rate in patients hospitalized with an NSTEMI in non-expansion states and expansion states based on an interrupted time series (ITS) model after adjusting patient-level and hospital effects, without imposing restrictions on time trends and making no assumptions that time effects were different before or after 2014. The solid lines are based instead on a comparative interrupted time-series (CITS) model which assumes that pre-intervention and post-intervention trends are linear in order to interpret changes in revascularization rates before and after Medicaid expansion.

This sensitivity analysis was conducted after excluding patients from Washington, D.C., Delaware, Massachusetts, New York, and Vermont

**Appendix Figure 14.** The solid circles represent the yearly estimates for the revascularization rate in patients hospitalized with a STEMI based on an interrupted time series (ITS) model after adjusting patient-level and hospital effects, without imposing restrictions on time trends and making no assumptions that time effects were different before or after 2014. The solid line is based on a comparative interrupted time-series (CITS) model which assumes that pre-intervention and post-intervention trends are linear in order to interpret changes in revascularization rates before and after the ACA.

**Appendix Figure 15.** The solid circles represent the yearly estimates for the revascularization rate in black and white patients hospitalized with a STEMI based on an interrupted time series (ITS) model after adjusting patient-level and hospital effects, without imposing restrictions on time trends and making no assumptions that time effects were different before or after 2014. The solid lines are based instead on a comparative interrupted time-series (CITS) model which assumes that pre-intervention and post-intervention trends are linear in order to interpret changes in revascularization rates before and after Medicaid expansion.
